# Infection with human cytomegalovirus, Epstein-Barr virus, and high-risk types 16 and 18 of human papillomavirus in *EGFR*-mutated lung adenocarcinoma

**DOI:** 10.3325/cmj.2023.64.84

**Published:** 2023-04

**Authors:** Suzana Harabajsa, Hajdi Šefčić, Marija Klasić, Marija Milavić, Snježana Židovec Lepej, Ivana Grgić, Matea Zajc Petranović, Marko Jakopović, Silvana Smojver-Ježek, Petra Korać

**Affiliations:** 1 Department for Pathology and Cytology, Division of Pulmonary Cytology Jordanovac, University Hospital Center Zagreb, Zagreb, Croatia; 2 Department for Biology, Division of Molecular Biology, Faculty of Science, University of Zagreb, Zagreb, Croatia; 3 Institute of Pathology, School of Medicine, University of Zagreb, Zagreb, Croatia; 4 Department of Immunology and Molecular Diagnostics, University Hospital for Infectious Diseases Zagreb, Zagreb, Croatia; 5 Institute for Anthropological Research, Zagreb, Croatia; 6 Department for Lung Diseases Jordanovac, University Hospital Center Zagreb, Zagreb, Croatia; 7 School of Medicine, University of Zagreb, Zagreb, Croatia

## Abstract

**Aim:**

To assess the frequency of human cytomegalovirus (HCMV), Epstein-Barr virus (EBV), and high-risk types of human papillomavirus (HPV16 and HPV18) infections in lung adenocarcinoma samples.

**Methods:**

Lung adenocarcinoma cytological smears and their DNA isolates were obtained from patients hospitalized at the Department for Lung Diseases Jordanovac, Zagreb, in 2016 and 2017. Overall, 67 lung adenocarcinoma samples were examined: 34 with epidermal growth factor receptor gene (*EGFR*) mutations and 33 without *EGFR* mutations. The *EGFR* mutation status and virus presence were assessed with a polymerase chain reaction, and random samples were additionally tested for EBV with Sanger sequencing. HCMV, EBV, HPV16, and HPV18 infections were evaluated in relation to *EGFR* mutation, smoking status, and sex. A meta-analysis of available data about HPV infection in non-small cell lung cancer was performed.

**Results:**

More frequent HCMV, EBV, HPV16, and HPV18 infections were observed in lung adenocarcinoma samples with *EGFR* mutations than in samples without these mutations. Coinfection of the investigated viruses was observed only in lung adenocarcinoma samples with mutated *EGFR*. In the group with *EGFR* mutations, smoking was significantly associated with HPV16 infection. The meta-analysis showed that non-small cell lung cancer patients with *EGFR* mutations had a higher odds of HPV infection.

**Conclusion:**

HCMV, EBV, and high-risk HPV infections are more frequent in *EGFR*-mutated lung adenocarcinomas, which indicates a possible viral impact on the etiology of this lung cancer subtype.

Lung cancer is the leading cause of cancer-related death in both sexes (1,2). Tobacco use is considered a major risk factor; however, 10%-15% of cases occur in non-smokers (3). Lung cancer is also linked to environmental factors, toxic and infectious agents, and viruses. Viruses and other infectious agents cause nearly 20% of all cancers in humans. Viral infection and the consequent integration of the viral DNA can alter the tumor microenvironment (2). The expression of viral genes can change the activity of cellular proto-oncogenes or tumor suppressor genes, which generally promotes oncogene activity (4). Viral integration into the host genome can alter host gene expression by disrupting its sequence (5-7). Non-small cell lung cancer (NSCLC) represents 85% of all lung cancers and is subcategorized into three main groups: lung adenocarcinoma (LA), squamous cell carcinoma (SqCC), and large-cell carcinoma. In small biopsies and cytology specimens,10%-30% of NSCLCs are morphologically diagnosed as “not otherwise specified” (NSCLC-NOS) (1,8-10). Lung cancer in non-smokers is associated with mutations in the epidermal growth factor receptor gene (*EGFR*) (3,8,11). The link between NSCLC and the most common viruses, such as human cytomegalovirus (HCMV), Epstein-Barr virus (EBV), and high-risk types 16 and 18 of human papillomavirus (HPV16/18) is still under investigation (12). These viruses are considered oncogenic and are associated with different types of tumors (13). HCMV, EBV, and HPV16/18 may also be involved in the development of lung cancer, but the results are inconsistent or reflect geographic variations (12,13). HCMV is a DNA β-herpesvirus capable of infecting various types of human cells and is mainly associated with glandular cell types of cancers such as adenocarcinoma (14). This virus infects 80% of the adult human population (4,6) and is able to establish lifelong persistence and latent infection after primary exposure. HCMV infection prevents external signaling to the cell by disrupting *EGFR* function, which is essential for HCMV binding, signaling, and entry into the host cell (15). EBV is a lymphotropic DNA virus that infects more than 90% of adults worldwide, and its presence has been reported in LA and SqCC (16,17). HPV is an epitheliotropic DNA virus. HPV16/18 genotypes were most frequently detected in squamous cell types of cancers and were associated with non-smoking lung cancer (18-25). Viral carcinogenesis is well documented in at least 20% of human cancers, but our understanding of viral involvement in lung cancer pathogenesis is insufficient (13). The aim of this study was to assess the frequency of EBV, HCMV, and high-risk HPV infection, and the coinfection of these viruses, in the adenocarcinoma subtype of NSCLC with and without *EGFR* gene mutations. The association of these viruses with smoking status, age, and sex in Croatian population was also assessed. Furthermore, we performed a meta-analysis of HPV prevalence in patients with NSCLC and *EGFR* mutations compared with patients with NSCLC and without *EGFR* mutations from seven populations worldwide (Supplemental Material(Supplementary material)).

## Patients and methods

Sixty-seven LA cytological smears and their DNA isolates were obtained from patients hospitalized at the Department for Lung Diseases Jordanovac in 2016 and 2017. The study was approved by the Ethics Committee of Zagreb University Hospital Center.

### Cytological smear processing

**Figure 1:**
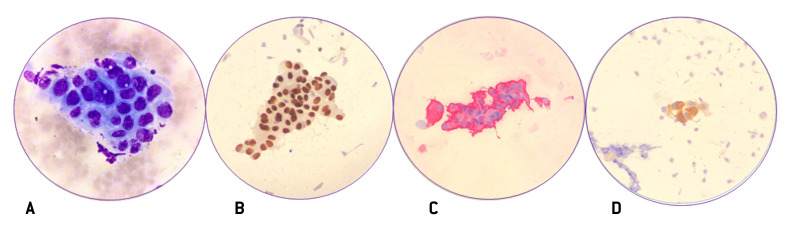
Lung adenocarcinoma cytomorphology: (**A**) Cellular clusters with acinar or papillary-like arrangement, uniform cells with eccentric, large, and round nuclei. Chromatin is fine, nucleoli are prominent. The cytoplasm is moderate to abundant, basophilic, and foamy or with large vacuoles and mucin production (May Grünwald Giemsa staining, magnification 1000 × ); (**B**) Thyroid transcription factor-1 nuclear positivity (magnification 400 × ); (**C**) Cytokeratin 7 cytoplasmic positivity (magnification 400 × ); (**D**) Napsin A cytoplasmic positivity (magnification 400 × ).

The LA cytological smears were made from bronchial washings (n = 15), bronchial brushings (n = 15), small biopsy imprints (n = 13), and transbronchial fine-needle aspirations (n = 4) obtained during bronchoscopies. LA cytological smears made from transthoracic fine-needle aspirations (n = 3), lymph node fine-needle aspirations (n = 1), and pleural effusions (n = 16) were also obtained. All cytological samples were routinely processed and analyzed. The liquid samples of bronchial washings and pleural effusions were first centrifuged and cytocentrifuged, while others were directly applied in a thin layer to cytological slides in order to obtain good-quality smears with a sufficient number of malignant cells. In the case of poorly differentiated lung cancer cells, immunocytochemistry (ICC) anti-cytokeratin 7 (CK7, clone OV-TL12/30; Dako Glostrup, Denmark), anti-thyroid transcription factor-1 (TTF-1, clone 8G7G3/1TTF-1; Dako), and anti-napsin A (napsin A; clone KCG1.1; Abcam, Cambridge, UK) were applied to distinguish LA from SqCC and NSCLC-NOS. Typical ICC features of LA were TTF-1 nuclear positivity and CK7 and napsin A cytoplasmic positivity (1,8) (Figure 1). The May Grünwald Giemsa-stained smears with a minimum of 30% cells and/or more than 400 LA cells were tested for *EGFR* mutations. The studied *EGFR* gene mutations were classical (ex19del; L858R) and rare (G719X; ex20ins; S768I; T790M; ex21).

### EGFR gene mutation and virus detection

DNA was extracted from all cytological smears (cells were scraped from each slide) with a Cobas DNA Sample Preparation Kit (Roche, Basel, Switzerland) without the deparaffinization procedure. DNA was quantified with a NanoDrop ND-1000 spectrophotometer (Thermo Fisher Scientific, Waltham, MA, USA). The Cobas® EGFR Mutation Test v2 and Cobas z 480 real-time PCR analyzer (both from Roche) were used for automated amplification and detection of mutations in the *EGFR* gene. Virus detection was performed with previously described PCR-based protocols (26-30). The presence of HCMV DNA was detected by amplifying two regions: a region within the major immediate-early gene (*MIE*) and a region located within glycoprotein B (*gB*) (26,27). The presence of EBV DNA was detected by amplifying the sequence within the Bam H1W region (30). The presence of HPV16 and HPV18 DNA was detected by amplifying a fragment of the *E7* gene (29) (Table 1). DNA isolated from samples confirmed to contain specific viral DNA was used as a positive control (Table 2). All amplifications for HCMV, EBV, HPV16, and HPV18 were carried out for 40 cycles at a denaturing temperature of 98 °C for 10 s, an annealing temperature of 60 °C for 30 s, and a primer extension temperature of 72 °C for 30 s.

**Table 1:** Primer sequences for detection of human cytomegalovirus (HCMV), Epstein-Barr virus (EBV), and human papillomavirus (HPV) types 16 and 18*

Virus	Primer	Primer sequence (5′ - 3′)*	Product size (bp)
HCMV	MIE6	AGTGTGGATGACCTACGGGCCATCG	110
	MIE7	GGTGACACCAGAGAATCAGAGGAGC	
	CMV 5A	TCATGAGGTCGTCCAGA	326
	CMV 5B	TGAGGAATGTCAGCTTC	
	CMV 5C	TCGTCCAGACCCTTGAGGTA	301
	CMV 5D	CCAGCCTCAAGATCTTCAT	
EBV	EBV1	AAAGCGGGTGCAGTAACAGGTAAT	314
	EBV2	TTGACTGAGAAGGTGGCCTAGCAA	
HPV16	HPV16f	TGAGCAATTAAATGACAGCTCAGAG	212
	HPV16r	TGAGAACAGATGGGGCACACAAT	
HPV18	HPV18f	GACCTTCTATGTCACGAGCAATTA	236
	HPV18r	TGCACACCACGGACACACAAAG	

*Primer sequences were taken from previous studies (26-29).

**Table 2:** Samples used as positive controls for viral polymerase chain reaction-testing*

Virus	Sample
Human cytomegalovirus	Blood of a patient infected with the virus
Epstein-Barr virus	Lymphoma FFPE tissue sample
Human papillomavirus type 16	HeLa cell line
Human papillomavirus type 18	Cytological smear of a patient with cervical cancer

*Abbreviations: FFPE – formalin fixed paraffin embedded.

Due to the unexpected PCR results, random samples were additionally tested for EBV infection by using amplification followed by Sanger sequencing. The C terminus of the latent membrane protein 1 gene (*LMP1*) was amplified by using OF 8081: 5′-GCTAAGGCATTCCCAGTAAA-3′ and OR 8744: 5′-GATGAACACCACCACGATG-3′ as outer primers, and IF 8213: 5′-CGGAACCAGAAGAACCCA-3′ and IR 8719: 5′ -TCCCGCACCCTCAACAAG-3′ as inner primers (30,31). The PCR reactions were carried out in 45 cycles at 94 °C for 30 s, 60 °C for 30 s, and 72 °C for 1 min with FastStart^TM^ High Fidelity (Sigma-Aldrich, Taufkirchen, Germany). The sequencing reaction was prepared by using inner primers (Macrogen Inc., Seoul, Korea) and the BigDye^TM^ Terminator v.3.1 Sequencing Kit (Thermo Fisher Scientific), and the segment was amplified for 25 cycles at 96 °C for 10 s, at 50 °C for 5 s, and at 60 °C for 4 min. Capillary electrophoresis of the sequencing products was carried out with an ABI PRISM^®^ 3500 Genetic Analyzer (Thermo Fisher Scientific). The obtained sequences were analyzed and aligned with sequence accession numbers from MK 507915 to MK507954 with Vector NTI Software (Thermo Fisher Scientific).

### Meta-analysis

The primary search generated 34 potentially relevant articles, six of which met the inclusion criteria (32-37) (Supplemental Material(Supplementary material)). Two studies (38,39) did not find HPV in NSCLC patients whether or not they had EGFR mutations, and therefore could not be included in the meta-analysis.

### Statistical analysis

Differences between the groups were assessed with a Mann-Whitney U test and Fisher exact test, while the association between categorical variables was evaluated with a χ^2^ test. The significance level was set at *P* < 0.05. The statistical analysis was performed with STATISTICA 10 (Tibco, Palo Alto, CA, USA) and STATA 17 (StataCorp, College Station, TX, USA).

## RESULTS

There were 34 LA patients with *EGFR* mutations (20 or 58.8% women) and 33 LA patients without *EGFR* mutations (20 or 60.6% women). The median age of both groups was 69 years. Among LA patients with *EGFR* gene mutations, 24 were non-smokers, eight were smokers (including ex-smokers), and two had unavailable data on smoking status. Among patients without *EGFR* gene mutations, 23 were smokers (including ex-smokers) and 10 were non-smokers.

### HCMV, EBV, HPV16, and HPV18 infection

The HCMV *MIE* gene was significantly more frequently detected in LA samples with *EGFR* gene mutations than in LA samples without *EGFR* mutations (14/34 vs 0/33, *P* < 0.001, Table 3). The HCMV *gB* was more frequently detected in LA samples with *EGFR* gene mutations than in samples without *EGFR* mutations (4/34 vs 1/33), but the difference was not significant. EBV (26/34 vs 6/33, *P* < 0.001), HPV16 (11/34 vs 3/33, *P* < 0.05), and HPV18 (24/34 vs 1/33, *P* < 0.001, Table 3) were significantly more frequently detected in LA samples with *EGFR* gene mutations than in LA samples without *EGFR* mutations. HPV16 infection was significantly associated with smoking in LA patients with *EGFR* mutations (*P* = 0.028). HCMV, EBV, and HPV18 infection was not significantly associated with age, sex, and smoking status in LA patients with *EGFR* gene mutations.

**Table 3:** Viral infections in lung adenocarcinoma samples*^†‡^

	Lung adenocarcinoma with *EGFR* gene mutations^‡^					Lung adenocarcinoma without *EGFR* gene mutations				
	men	women	smokers	non-smokers	unknown smoking status	men	women	smokers	non-smokers	unknown smoking status
HCMV *MIE*	4	10	1	11	2	0	0	0	0	0
HCMV *gB*	2	2	3	1	0	0	1	0	1	0
EBV	11	15	7	17	2	4	2	5	1	0
HPV16	6	5	5	6	0	2	1	1	2	0
HPV18	9	15	6	16	2	0	1	1	0	0

*Abbreviations: *EGFR* – epidermal growth factor receptor gene; HCMV – human cytomegalovirus; *MIE* – major immediate-early gene; gB – glycoprotein B; EBV – Epstein-Barr virus; HPV – human papillomavirus type.

†Viral infection of all tested viruses was significantly more common in patients with lung adenocarcinoma containing *EGFR* mutations than in those without *EGFR* mutations.

‡All samples of lung adenocarcinoma patients with *EGFR* gene mutations tested positive for more than one analyzed virus.

### Viral coinfections

All samples of LA patients with *EGFR* gene mutations showed coinfection of analyzed viruses. The most frequent coinfection was that of EBV and HPV18 (in 19 samples). The coinfection of EBV and HCMV was detected in 11 samples and the coinfection of EBV and HPV16 in eight samples. There were no coinfections in LA patients without *EGFR* mutations. The prevalence of viral coinfections in LA patients with *EGFR* gene mutations was significantly higher than that in LA patients without *EGFR* mutations (*P* < 0.001).

### Confirmation of test accuracy

*EBV detection by Sanger sequencing*. All LA samples with *EGFR* gene mutations confirmed by PCR to be positive for a particular virus showed one amplicon corresponding to a particular virus fragment of the expected size and several additional ones of different sizes. Samples without *EGFR* gene mutations, when positive for a particular virus, in most cases showed only an amplicon of the expected size. However, positive controls always showed only one amplicon of the expected size. In order to identify additional amplicons in LA samples with *EGFR* gene mutations, we used EBV detection based on Sanger sequencing. After amplification of randomly selected samples and gel electrophoresis, all samples were isolated from the gel and sequenced. The sequences were 206, 461, and 534 nucleotides long, and aligned with 40 other C-terminus sequences of the *LMP1* gene. The two longer sequences matched the available EBV *LMP1* gene sequences in length; however, the alignment was incomplete, and less than 50% of nucleotides matched the sequences with the accession numbers MK507915 to MK507954.

**Figure 2:**
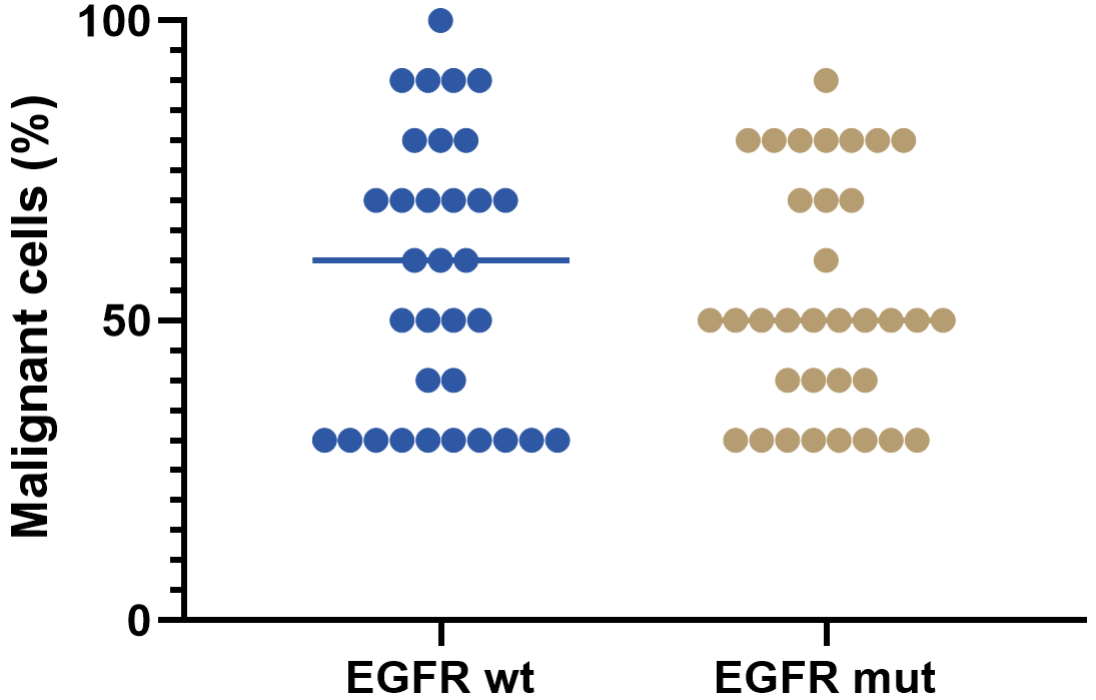
The percentage of malignant cells per sample did not differ between the two subgroups of lung adenocarcinoma patients based on epidermal growth factor receptor (*EGFR*) gene mutation status. Only cytological smears with a minimum of 30% cells and/or more than 400 cells of lung adenocarcinoma were considered adequate for *EGFR* gene mutation testing by polymerase chain reaction method. Abbreviations: mut – mutated; wt – wild type.

*HCMV, EBV, HPV16, and HPV18 infection in relation to a different percentage of cancer cells and DNA concentration per sample.* We assessed if there was a difference in viral detection based on cancer cell percentage and DNA concentration in LA with or without *EGFR* gene mutations. Slightly lower mean concentrations of DNA were noticed in LA smears with a higher percentage of cancer cells, but the difference was not significant (Supplemental Table 1(Supplementary Table 1)) (Figure 2).

*HCMV, EBV, HPV16, and HPV18 infection in different types of lung adenocarcinoma samples.* To rule out differences in viral detection between smears prepared from liquid samples or samples directly applied to cytological slides in LA samples with *EGFR* gene mutations, we evaluated the correlation between them and PCR-confirmed infections. A lower percentage of cancer cells was noticed in smears prepared from liquid samples but the difference was not significant (Supplemental Table 2(Supplementary Table 2)).

### Meta-analysis of HPV prevalence in NSCLC

Meta-analysis confirmed a higher prevalence of HPV among lung cancer patients with *EGFR* mutations (Supplemental Material(Supplementary material)).

## DISCUSSION

In this study, HPV infection was present in 43% of LA samples. In previous studies, the average prevalence of different types of HPV in the European population was 16%, but in Asia it averaged 37% (24). The average HPV prevalence in America was 12.5% and the figure in Australia was 18.5% (24). In a recent Greek study (25), HPV was found in only 3% of the samples, and an Indian study showed HPV prevalence to be 6.8% (12). In our study, significantly more frequent HPV infection was found in LA samples with *EGFR* gene mutations than in samples without *EGFR* gene mutations, which agrees with previous research (37). The reason for more frequent HPV infection in LA with *EGFR* gene mutations could be the viral protein E6. This protein upstream regulates the inhibitors of apoptosis of the EGFR/PI3K/AKT signaling pathway and may play a central role in NSCLC cancer tumorigenesis with *EGFR* gene mutations and HPV infection (40,41). Previous studies have confirmed HPV as part of the etiology of cervical and oropharyngeal cancer, but the association with lung cancer has not been fully established (12,25). Discrepancies on the subject are mainly attributed to high heterogeneity among studies, geographical variations in epidemiological risk factors, and different methods of viral DNA detection.

EBV and HCMV infections in our study were also detected at a higher percentage than expected. EBV was found in 47.8% of LA samples. In previous European studies, EBV was found in lung cancer cells in a very small percentage or was completely absent, but in Asian studies, its incidence was between 30% and 50% (12). EBV was detected in a condensed exhaled concentrate from about 30% of lung cancer patients and from 20% of controls (42). Our study showed significantly more frequent EBV infection in LA samples with *EGFR* gene mutations than in samples without *EGFR* gene mutations. The literature data about the association between lung cancer and EBV are scarce. The high percentage of EBV infections in our study could be explained by the fact that EBV infection is quite common, and 90% of the population has latent infection with a small number of viral copies over a lifetime. Yet, latent infection with a small number of viral copies is mainly retained in memory B-lymphocytes, and EBV was not detected or was detected in a lower percentage of healthy seropositive individuals. However, it was detected in a large percentage of samples isolated from Burkitt's lymphoma or Hodgkin's lymphoma (42,43). The results of previous studies of EBV infection in lung cancer are inconsistent, and the role of EBV in tumorigenesis remains unclear. To confirm our results, DNA sequencing was performed on randomly selected samples and the results were in line with the PCR results (44). The mechanisms through which EBV acts via *EGFR* are not fully explained. It is possible that, like HPV or HCMV, it alters the regulation of one of the signaling pathways acting via *EGFR* and is important for the life cycle and survival of cells. To date, the LMP2A protein in EBV has been proposed to increase the metastatic potential of cancer cells by accelerating integrin cleavage (45).

HCMV is also widespread worldwide and was detected in 28% of LA samples in our study. Of these, HCMV *MIE* was detected in 41% and HCMV *gB* in 13% of the samples. A significant difference in the prevalence of HCMV *MIE* was found between LA samples with *EGFR* gene mutations and LA samples without *EGFR* mutations, while no such difference was found in the prevalence of HCMV *gB*. A French and an Indian HCMV study in lung cancer found no positive cases. At the same time, an Italian study found 10% of positive cases, but there was no significant difference between cancer patients and healthy individuals (42). The prevalence of HCMV *MIE* obtained in our study corresponds to the results of a study that used the same set of primers to detect the *MIE* gene (46). In that study, HCMV *MIE* was detected in 23% of participants and was not detected in the control group. The mentioned study did not investigate cancers with *EGFR* mutations, so the number of participants with *EGFR* mutations remained unknown (46). Significantly more frequent HCMV *MIE* infection in LA with *EGFR* mutations could be explained by HCMV using the EGFR-associated signaling pathway as a receptor to enter the cell and thus acting as an oncogene inhibiting the protein p53 via the *MIE* gene (47,48). By disrupting the immune system, HCMV could moderate the response to therapy (49). We simultaneously analyzed the *MIE* and *gB* gene regions of HCMV to avoid false-negative results. Both the MIE and gB HCMV proteins play an essential role in the viral entry into the host cell, while the gB protein additionally plays an important role in the replication cycle of HCMV (48). Therefore, parallel detection of HCMV based on *MIE* and *gB* fragments would be expected, but this was not the case in our study.

Another interesting finding of our study was viral coinfection. The most common coinfection in LA with *EGFR* mutations was EBV in combination with HPV, followed by HCMV. Due to the relatively small number of samples used in our study, we planned to perform a meta-analysis of HCMV, EBV, and HPV infections in NSCLC with *EGFR* mutations, but the existing data allowed this only for HPV. Our meta-analysis (Supplemental Material(Supplementary material)) showed that NSCLC patients with *EGFR* mutations had higher odds of HPV infection than NSCLC patients without *EGFR* mutations, regardless of their ethnicity. The same finding was reported in a meta-analysis that included four Asian populations (50).

Although our study was conducted on a relatively small number of LA samples, the obtained results point to a possible viral influence on the etiology of LA with *EGFR* mutations.

Significant differences in infection rates indicate that lung adenocarcinoma with *EGFR* gene mutations and lung adenocarcinoma without *EGFR* gene mutations have a different viral background, and that HCMV, EBV, and high-risk types 16 and 18 of HPV may therefore affect the etiology of EGFR-mutated lung adenocarcinoma.
