# Supplementary material for: Infection with human cytomegalovirus, Epstein-Barr virus, and high-risk types 16 and 18 of human papillomavirus in EGFR-mutated lung adenocarcinoma
Source: Croat Med J. 2023 Apr;64(2):84–92. doi: 10.3325/cmj.2023.64.84 (PMC10183960; doi:10.3325/cmj.2023.64.84)

SUPPLEMENTAL TABLE. Characteristics of the seven studies included in the meta-analysis of the HPV prevalence in NSCLC*

Publication	N	Sex f/m	Age range	NSCLC subtype	EGFR mutation status	HPV detection method	HPV positive	Population	Country
This study	67	40/27	39-89 median 69 yrs	64 LA, 3 NSCLC-NOS	34/67	PCR	45/67	Caucasian	Croatia
Marquez Medina et al 2013	40	20/20	38-80	5 SQCC, 23 LA, 4 LA+BAC, 8 NSCLC-NOS	6 unknown, 23 negative, 11 with mutations	PCR	1/34	Caucasian	Spain
Sagerup et al 2014	336	169/167	33.9-84.1	219 LA, 87 SQCC, 7 LCLC, 4 SCLC, 14 carcinoids, 2 adenosquamous, 3 others	26/328	PCR	13/336	Caucasian	Norway
Kato et al 2012	42	14/28	17 were <70 yrs, and 25>70y-old	26 LA, 12 SQCC, and 4 others	13/42	PCR	7/42	Asian	Japan
Tung et al 2013	151	53/98	NA	58 SQCC, 93 LA	45/151	IHC	68/151	Asian	Republic of China (Taiwan)
Wang et al 2014	210	108/102	median 69.5 yrs; 108 were <70 y-old, 102 were >70 y-old	LA	88/210	nested PCR	74/210	Asian	Republic of China (Taiwan)
Li et al 2016	95	44/51	50 were <64 y-old, 45 >64y-old	advanced LA	44/95	PCR	27/95	Asian	Republic of China

*Quantitative variables for meta-analysis are presented in absolute numbers.

†Complete references of the study are given in the References.

Abbreviations: LA: lung adenocarcinoma; BAC: bronchoalveolar carcinoma; IHC: immunohistochemistry; LCLC: large cell lung carcinoma; NSCLC: non-small cell lung carcinoma; NSCLC-NOS: non-small cell lung carcinoma-not otherwise specified; SCLC: small cell lung carcinoma; SQCC: squamous cell carcinoma

SUPPLEMENTAL FIGURE 1. A flowchart of a selection process of studies eligible for the meta-analysis of HPV prevalence in NSCLC.

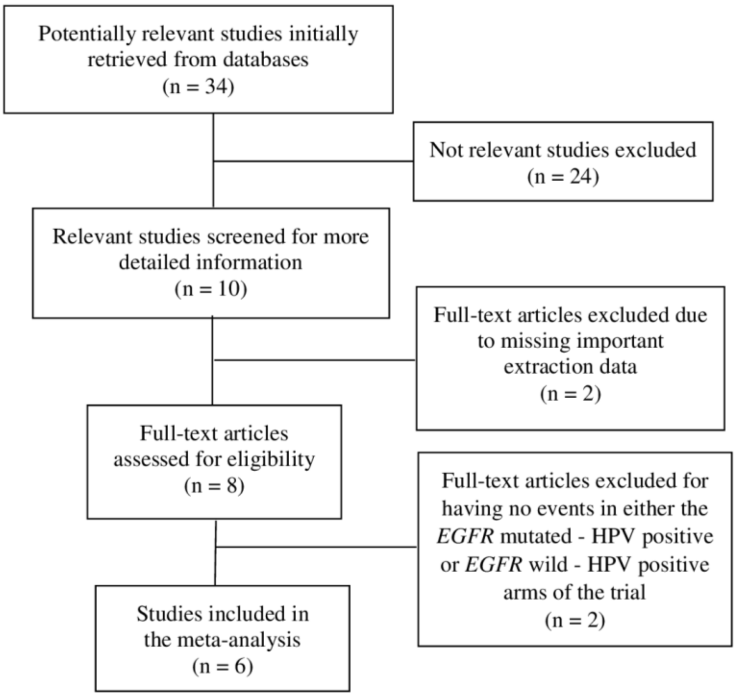

SUPPLEMENTAL FIGURE 2. Forest plot of the association between patients with NSCLC with *EGFR* mutations and odds ratio (OR) for HPV prevalence, compared with patients with NSCLC without *EGFR* mutations. The results were stratified by country and ethnicity.

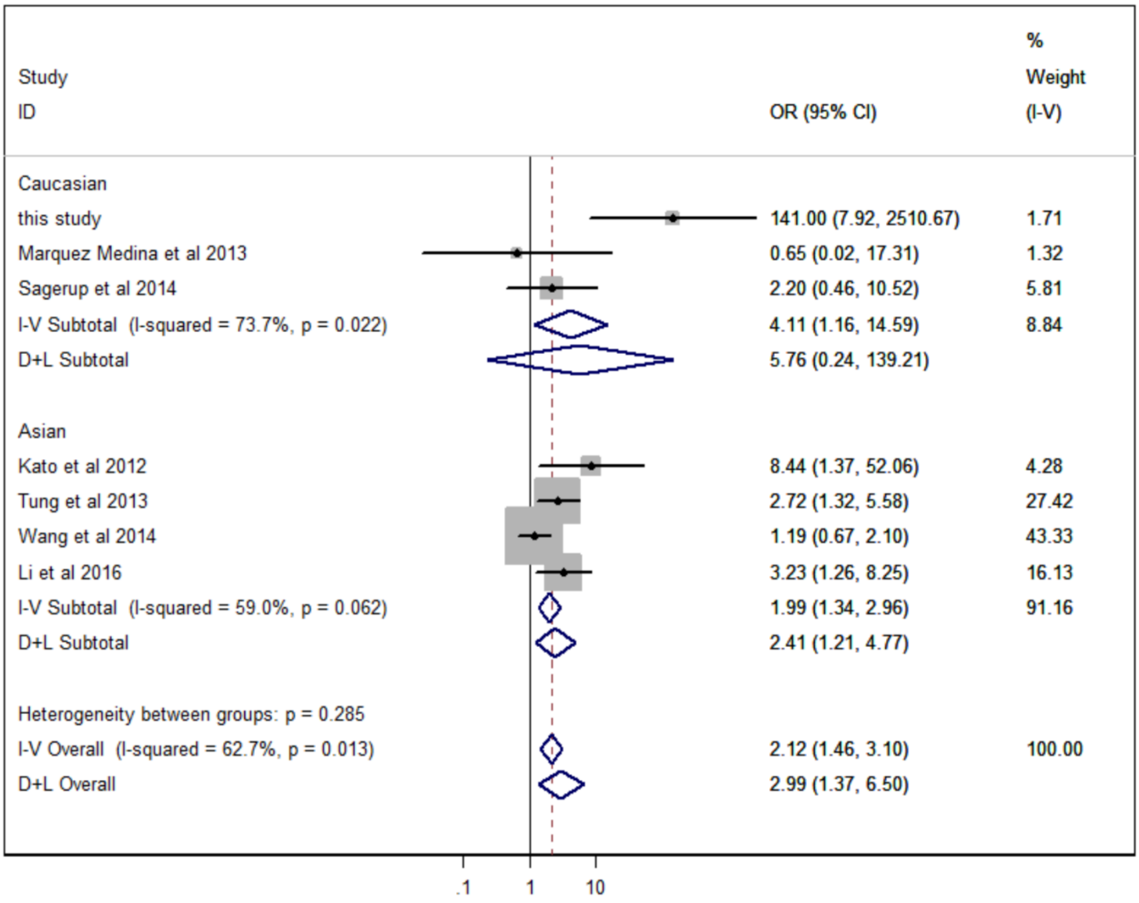

SUPPLEMENTAL FIGURE 3. Begg's funnel bias plot of studies included in the meta-analysis of HPV prevalence in NSCLC.

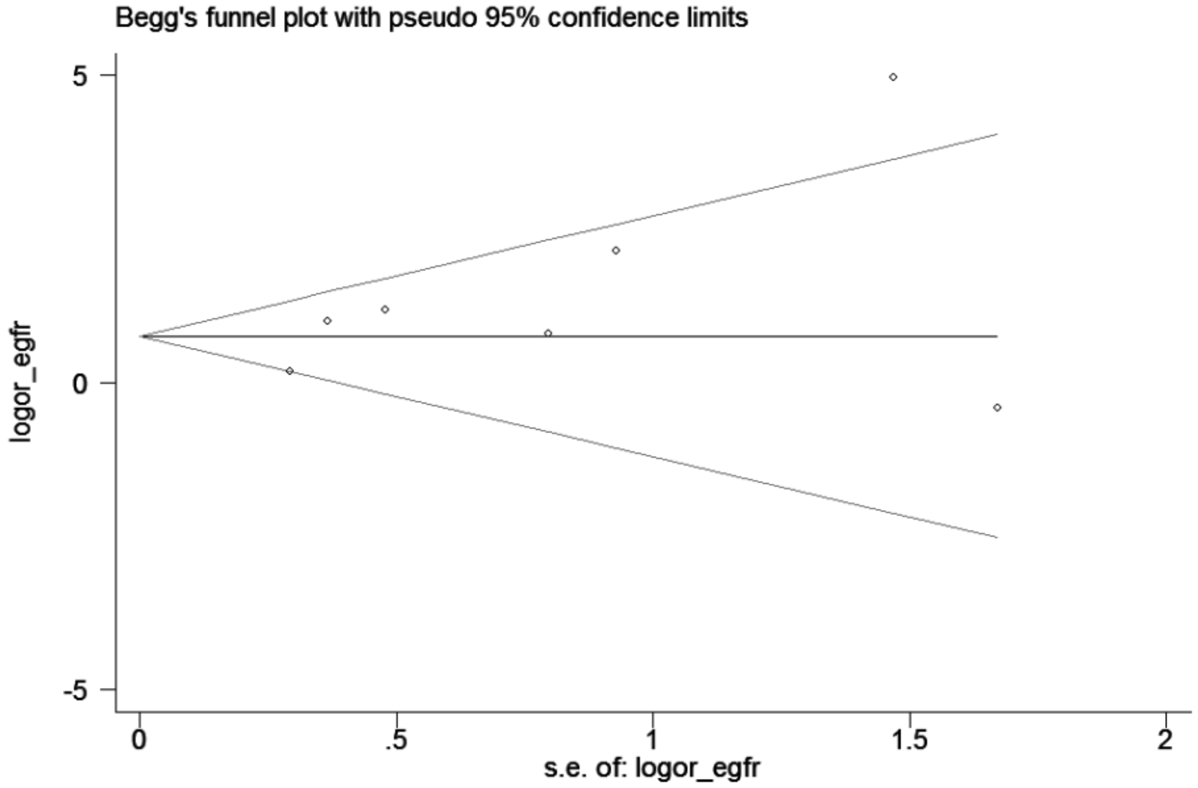

SUPPLEMENTAL FIGURE 4. Egger`s publication bias plot of studies included in the meta-analysis of HPV prevalence in NSCLC.

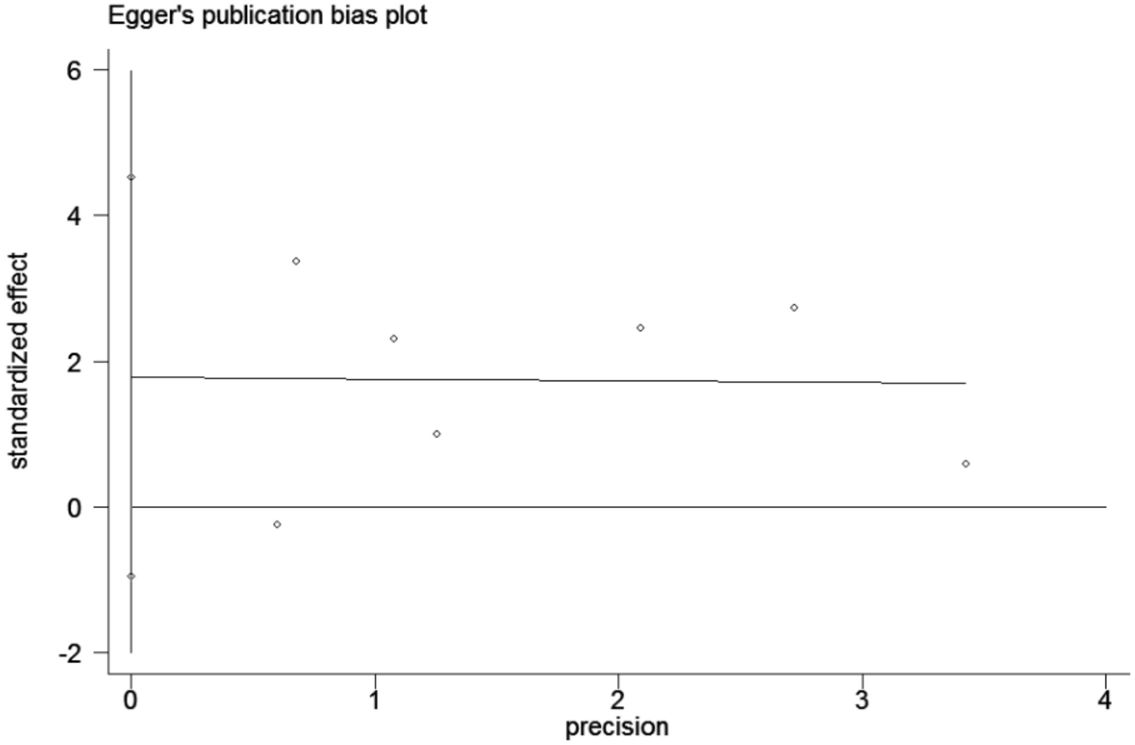

SUPPLEMENTAL FIGURE 5. The influential plot of NSCLC-HPV meta-analysis with the effects estimates (ORs) after omitting an individual study each time.

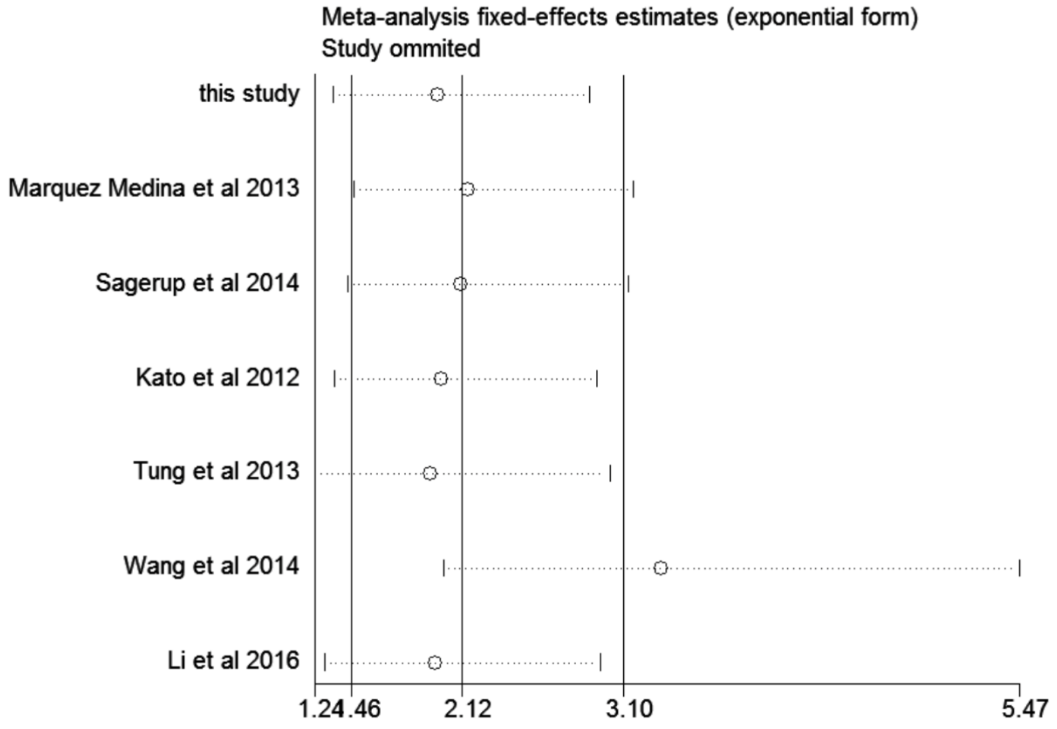

Supplement: Supplementary Material [file CroatMedJ_64_s003.pdf]
