# Supplementary material for: Infection with human cytomegalovirus, Epstein-Barr virus, and high-risk types 16 and 18 of human papillomavirus in EGFR-mutated lung adenocarcinoma
Source: Croat Med J. 2023 Apr;64(2):84–92. doi: 10.3325/cmj.2023.64.84 (PMC10183960; doi:10.3325/cmj.2023.64.84)
Supplement: Supplementary Table 1 [file CroatMedJ_64_s004.pdf]

SUPPLEMENTAL TABLE 1. HCMV, EBV, HPV16, and HPV18 infection in relation to a different percentage of cancer cells and DNA. concentration per sample.

		mean DNA concentration (ng/μL)	HCMV positive	EBV positive	HPV16 positive	HPV18 positive
Lung adenocarcinoma samples with *EGFR* gene mutations‡	30-60% cancer cells n = 21	16.01 (min 5.1; max 66.8)	11	16	6	14
	> 60% cancer cells n = 13	11.09 (min 5.3; max 36.7)	6	11	4	9
Lung adenocarcinoma samples without *EGFR* gene mutations	30-60% cancer cells n = 16	17.4 (min 5.9; max 89.1)	0	4	0	0
	> 60% cancer cells n = 17	14.04 (min 5.5; max 45.6)	1	2	3	1

HCMV – human cytomegalovirus; EBV – Epstein-Barr virus; HPV16 – human papillomavirus type 16; HPV18 - human papillomavirus type 18.
‡All samples of lung adenocarcinoma patients with *EGFR* gene mutations were positive for more than one analyzed virus.
