# Supplementary material for: Infection with human cytomegalovirus, Epstein-Barr virus, and high-risk types 16 and 18 of human papillomavirus in EGFR-mutated lung adenocarcinoma
Source: Croat Med J. 2023 Apr;64(2):84–92. doi: 10.3325/cmj.2023.64.84 (PMC10183960; doi:10.3325/cmj.2023.64.84)
Supplement: Supplementary Table 2 [file CroatMedJ_64_s005.pdf]

SUPPLEMENTAL TABLE 2. HCMV, EBV, HPV16 and HPV18 infection in different types of lung adenocarcinoma samples.

		HCMV positive	EBV positive	HPV16 positive	HPV18 positive
Lung adenocarcinoma samples with *EGFR* gene mutations‡	Liquid samples n = 16	8	11	3	10
	Non-liquid samples n = 18	9	15	7	13
Lung adenocarcinoma samples without *EGFR* gene mutations	Liquid samples n = 15	0	4	1	1
	Non-liquid samples n = 18	1	2	2	0

HCMV – human cytomegalovirus; EBV – Epstein-Barr virus; HPV16 – human papillomavirus type 16; HPV18 - human papillomavirus type 18.
‡All samples of lung adenocarcinoma patients with *EGFR* gene mutations were positive for more than one analyzed virus.
